# Perioperative Immunonutrition in Patients Undergoing Lung Cancer Surgery: Current Evidence and Future Perspectives

**DOI:** 10.3390/nu18142381

**Published:** 2026-07-21

**Authors:** Alicja Werblińska, Piotr Jerzy Skrzypczak, Magdalena Roszak, Maciej Bryl, Cezary Piwkowski, Piotr Gabryel

**Affiliations:** 1 Department of Thoracic Surgery, Poznan University of Medical Sciences, Szamarzewskiego 62 Street, 60-569 Poznan, Poland; piotr.j.skrzypczak@gmail.com (P.J.S.); cpiwkowski@wcpit.org (C.P.); 2 Doctoral School, Poznan University of Medical Sciences, Bukowska 70, 60-812 Poznan, Poland; 3 Greater Poznan Center of Pulmonology and Thoracic Surgery, Szamarzewskiego 62 Street, 60-569 Poznan, Poland; mbryl@wcpit.org; 4 Department of Pathophysiology, Poznan University of Medical Sciences, 61-701 Poznan, Poland; mmr@ump.edu.pl

**Keywords:** lung cancer, immunonutrition, thoracic surgery, perioperative care

## Abstract

**Background/Objectives**: Lung cancer remains the leading cause of cancer-related mortality worldwide, and surgical resection is the primary curative treatment for patients with early-stage non-small cell lung cancer (NSCLC). Patients undergoing lung cancer surgery are frequently affected by malnutrition, systemic inflammation, sarcopenia, and cancer-related cachexia, which may adversely affect postoperative recovery and clinical outcomes. Perioperative immunonutrition has been proposed as a strategy to support immune and metabolic responses associated with surgical stress. This narrative review summarizes current evidence regarding the role of perioperative immunonutrition in patients undergoing lung cancer surgery. **Methods**: This narrative review summarizes current evidence regarding perioperative immunonutrition in patients undergoing lung cancer surgery. Relevant studies evaluating perioperative immunonutrition, including formulations enriched with arginine, omega-3 fatty acids, glutamine, and nucleotides, were analyzed. Particular attention was given to clinical studies in thoracic surgical oncology, perioperative outcomes, inflammatory response, and current nutritional guideline recommendations. **Results**: Available evidence suggests that perioperative immunonutrition may improve nutritional and immunological status in patients undergoing lung cancer surgery. Clinical studies have reported reductions in postoperative complications, shorter chest drainage duration, improved nutritional indices, and decreased inflammatory markers in patients receiving immunonutritional support. Experimental and translational studies also indicate potential beneficial effects on immune cell function and inflammatory regulation. However, current thoracic-specific evidence remains limited because of small study populations, heterogeneity of nutritional protocols, and variability in study design. **Conclusions**: Perioperative immunonutrition appears to be a promising adjunct to comprehensive perioperative care in patients undergoing lung cancer surgery. Although preliminary evidence suggests potential benefits in postoperative recovery and nutritional optimization, its implementation should be individualized according to the patient’s nutritional status, disease stage, and overall treatment strategy. As immunonutrition modulates metabolic and immune pathways that may also influence tumor biology, nutritional interventions should be evidence-based, carefully monitored, and integrated within multidisciplinary perioperative care to maximize clinical benefits while minimizing potential unintended effects. Further large, well-designed randomized clinical trials are needed to establish standardized protocols and clarify the role of immunonutrition in thoracic surgical oncology.

## 1. Introduction

Lung cancer remains one of the most significant global health challenges. According to the latest epidemiological data, approximately 2,041,910 to 2,480,675 new cases and 1,817,469 to 618,120 deaths were recorded worldwide, including in the USA. Lung cancer is the leading cause of cancer-related mortality globally and one of the most frequently diagnosed malignancies [1]. Despite advances in diagnostics and treatment, the overall 5-year survival rate for lung cancer remains relatively low, estimated at approximately 28% [2].

Lung cancer is broadly classified into small-cell lung cancer (SCLC) and non-small-cell lung cancer (NSCLC), with NSCLC accounting for approximately 85% of all cases. Treatment strategies depend on several patient- and disease-specific factors, including tumor stage, histological subtype, and pulmonary function. Surgical resection remains the most effective curative treatment for patients with early-stage NSCLC [3].

Malnutrition and cancer-related weight loss are common among patients with lung cancer and represent important determinants of clinical outcomes. Studies suggest that up to 70% of patients experience involuntary weight loss during the course of the disease, often accompanied by progressive loss of skeletal muscle mass [3]. The development of malnutrition in cancer patients is multifactorial and is strongly associated with systemic inflammation and metabolic alterations induced by tumor-derived cytokines such as tumor necrosis factor-α (TNF-α), interleukin (IL)-1, and IL-6 [4]. These mediators contribute to muscle wasting, alterations in hepatic metabolism, and adipose tissue depletion [4].

Major oncologic surgery further exacerbates these metabolic disturbances. The surgical stress response is characterized by increased catabolism, hormonal changes, and activation of inflammatory pathways, all of which may impair immune function and worsen nutritional status. Consequently, malnourished patients are at higher risk of postoperative complications, prolonged hospital stay, and delayed recovery [5].

In this context, perioperative nutritional strategies aimed at modulating immune and metabolic responses have gained increasing attention. Immunonutrition refers to nutritional formulations enriched with one or more immunomodulating substrates provided in amounts exceeding those found in standard nutritional support. The most widely investigated and clinically used substrates include arginine, omega-3 fatty acids, nucleotides, and glutamine, although other bioactive nutrients with immunomodulatory properties have also been evaluated [6]. Although other nutrients, such as vitamin D and selected micronutrients, also possess immunomodulatory properties, they are generally not considered core components of immunonutrition formulations in the clinical nutrition literature. These nutrients may influence immune function, inflammatory responses, and tissue repair processes, potentially reducing surgery-related immunosuppression and improving postoperative outcomes [6,7].

Therefore, optimizing perioperative nutritional status, including the use of immunonutrition, may be an important component of comprehensive perioperative care in patients undergoing lung cancer surgery [5].

The aim of this review is to summarize current evidence regarding the role of perioperative immunonutrition in patients undergoing surgical treatment for lung cancer. Although perioperative immunonutrition has demonstrated promising effects in reducing postoperative complications and modulating inflammatory responses, the current evidence in patients undergoing lung cancer surgery remains inconclusive [8,9,10]. The limited number of randomized controlled trials, relatively small study populations, and methodological heterogeneity reduce the strength of existing recommendations [11,12]. Moreover, the impact of immunonutrition on long-term oncological outcomes, postoperative functional recovery, quality of life, and survival has not been sufficiently investigated [10]. The interaction between immunonutrition, cancer-associated cachexia, and modern multimodal perioperative care protocols also remains poorly understood [13]. These unresolved issues highlight the need for further well-designed prospective studies focused specifically on patients with NSCLC undergoing surgical treatment [14].

## 2. Materials and Methods

This narrative review summarizes current evidence on perioperative immunonutrition in patients undergoing lung cancer surgery. Relevant literature was identified through searches of PubMed, Embase, and the Cochrane Library up to May 2026. The search strategy included terms related to immunonutrition and thoracic oncology, including “immunonutrition”, “arginine”, “omega-3 fatty acids”, “glutamine”, “nucleotides”, “lung cancer”, “non-small cell lung cancer”, “thoracic surgery”, and “perioperative care”. Additional publications were identified through manual screening of reference lists from relevant articles. Priority was given to randomized controlled trials, clinical studies, systematic reviews, guideline documents, and selected translational studies addressing the role of immunonutrition in perioperative management of patients undergoing lung cancer surgery. Studies were selected based on their relevance to the topic and their contribution to the current understanding of immunonutrition in thoracic oncology.

## 3. Immunomodulating Nutrients and Their Biological Role in Perioperative Care

Perioperative immunonutrition aims to actively reshape the body’s metabolic and immunological response to surgical trauma, shifting the paradigm from basic caloric supplementation to targeted metabolic therapy [15]. Rather than merely correcting nutritional deficits, specialized formulas enriched with arginine, omega-3 fatty acids, glutamine, and nucleotides are designed to modulate both innate and adaptive immune responses, attenuate excessive systemic inflammation, preserve skeletal muscle mass, and promote postoperative recovery [16]. These mechanisms may help attenuate the metabolic and inflammatory response associated with major thoracic surgery. The trauma of lung resection triggers an immediate, profound catabolic state, marked by oxidative stress, compromised cellular immunity, and a chaotic cytokine cascade [16]. A summary of perioperative immunonutrients used in clinical practice is in Table 1.

**Figure 1:**
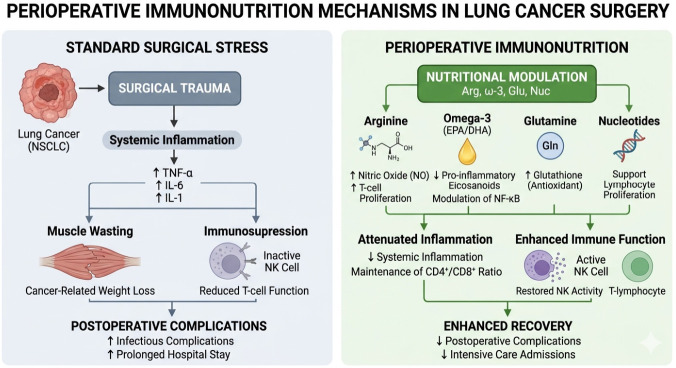
Biological pathways of standard surgical stress versus the immunomodulatory mechanisms of perioperative immunonutrition in lung cancer surgery. ↑ Increase; ↓ decrease.

In lung cancer patients, this surgical insult rarely occurs in isolation; it is routinely superimposed on baseline malnutrition, structural sarcopenia, and tumor-driven cachexia [15,31]. By intervening at the crossroads of nutrition and immunology, perioperative strategies aim to alter this destructive pathway and preserve postoperative functional reserves. While each immunonutrient operates through distinct biochemical pathways, their clinical value lies in their collective synergy—rebalancing immune responses, driving tissue repair, and preserving metabolic homeostasis when the patient’s body is under maximum stress [11]. The principal metabolic and immunological mechanisms through which perioperative immunonutrition modulates the response to surgical stress are summarized in Figure 1.

Current clinical evidence indicates that immunonutrition primarily modulates immune cell function rather than their circulating frequency. Clinical studies generally do not demonstrate consistent changes in peripheral neutrophil or monocyte counts [12]. Instead, improvements have been reported in functional parameters of innate immunity, including enhanced neutrophil phagocytic capacity, oxidative burst, chemotaxis, and microbial killing, as well as improved macrophage phagocytosis and, in experimental models, antigen presentation [32]. These functional changes are accompanied by modulation of inflammatory signaling, including reduced production of pro-inflammatory cytokines (TNF-α, IL-1β, and IL-6), increased IL-10 secretion, and attenuation of NF-κB activation. Experimental evidence further suggests that omega-3 fatty acids promote macrophage polarization toward a pro-resolving phenotype characterized by increased expression of CD206 and arginase-1 and reduced inducible nitric oxide synthase (iNOS), although these observations have been demonstrated predominantly in preclinical studies [33]. Overall, available evidence suggests that the immunological effects of perioperative immunonutrition are predominantly qualitative, improving immune cell function rather than altering the composition of circulating myeloid cell populations. Evidence regarding other myeloid cell populations remains scarce. Few clinical studies have evaluated eosinophils, basophils, or dendritic cell subsets, and no consistent quantitative changes have been reported. Likewise, changes in the overall myeloid-to-lymphoid ratio have not been systematically investigated in perioperative immunonutrition studies. Some clinical studies have reported postoperative reductions in the neutrophil-to-lymphocyte ratio (NLR), which are generally interpreted as reflecting attenuation of systemic inflammation rather than quantitative changes in circulating myeloid cell populations [6,34].

### 3.1. Arginine

Arginine is a semi-essential amino acid that plays an important role in immune regulation, wound healing, and nitrogen metabolism. Surgical trauma is associated with a transient period of immunosuppression, particularly affecting cellular immunity, which may increase susceptibility to infections and potentially promote tumor progression. Surgical stress induces systemic postoperative changes that negatively affect natural killer (NK) cell function, including the expansion of surgery-induced myeloid-derived suppressor cells (Sx-MDSCs) and a reduction in circulating arginine levels [35]. Experimental studies suggest that this temporary impairment of NK cell activity may increase the risk of postoperative metastatic spread, although most of the evidence comes from preclinical models. Arginine supplementation has been proposed as a strategy to counteract these effects by supporting immune cell function and facilitating immune recovery during the perioperative period [36].

One of the key mechanisms underlying the immunomodulatory properties of arginine is its role as a substrate for nitric oxide (NO) synthesis. Nitric oxide participates in multiple physiological processes, including the regulation of vascular tone, antimicrobial defense, and modulation of immune responses. In addition, arginine supports lymphocyte proliferation and enhances the activity of immune effector cells, particularly T lymphocytes and NK cells, which are essential for effective antitumor immunity [20].

The role of arginine in cancer biology is, however, complex. Besides supporting immune cell function, arginine also serves as a substrate for protein synthesis, polyamine production, and nitric oxide metabolism, pathways that may contribute to tumor cell proliferation depending on the metabolic phenotype of the tumor [19]. Conversely, arginine depletion within the tumor microenvironment, largely mediated by arginase-1-expressing myeloid-derived suppressor cells, suppresses T-cell activation and impairs antitumor immunity. Consequently, perioperative arginine supplementation is considered to restore immune competence in patients experiencing surgery-induced arginine depletion rather than to promote tumor growth, although further studies evaluating long-term oncological outcomes are warranted [37].

Beyond its immunological effects, arginine contributes to collagen synthesis and tissue repair through its role in polyamine and proline production, which are essential for wound healing and postoperative tissue regeneration. These properties may further support recovery following major surgical procedures [38].

In clinical practice, arginine is most commonly administered as part of specialized immunonutrition formulas rather than as a single nutrient. In perioperative studies, typical arginine intake provided through immunonutrition ranges from approximately 0.5 to 1.5 g/kg/day, usually administered for 5–10 days before surgery and, in some protocols, continued during the early postoperative period. Such regimens aim to optimize immune and metabolic responses before surgical stress [12,17,18].

Available evidence suggests that short-term perioperative arginine supplementation is generally safe and well tolerated when administered as part of standardized immunonutrition formulas. Current data do not indicate an increased risk of tumor growth or progression associated with perioperative arginine administration. Nevertheless, caution is recommended in patients with severe renal or hepatic insufficiency, in whom amino acid metabolism may be impaired. As with all nutritional interventions, supplementation should be individualized and integrated into a comprehensive perioperative nutritional strategy [12].

### 3.2. Omega-3 Fatty Acids

Omega-3 (ω-3) fatty acids, particularly long-chain polyunsaturated fatty acids (LC-PUFAs) such as eicosapentaenoic acid (EPA) and docosahexaenoic acid (DHA), represent an important component of immunonutrition in oncology. Their biological effects primarily involve modulation of the inflammatory response by influencing eicosanoid metabolism and the production of inflammatory mediators. EPA and DHA compete with ω-6 fatty acids for the same enzymatic pathways, including cyclooxygenase and lipoxygenase, leading to reduced synthesis of pro-inflammatory prostaglandins and leukotrienes and increased production of specialized pro-resolving mediators such as resolvins and protectins. These mechanisms may attenuate systemic inflammation associated with cancer and modulate the tumor microenvironment [39].

Experimental studies have also shown that ω-3 fatty acids may influence several signaling pathways involved in carcinogenesis, including NF-κB, PI3K/Akt, and STAT3. Through these mechanisms, ω-3 fatty acids may inhibit tumor cell proliferation, promote apoptosis, and modulate inflammatory signaling pathways involved in cancer progression [40].

The dietary ratio of ω-6 to ω-3 fatty acids has also been suggested to influence cancer risk. Epidemiological studies and Mendelian randomization analyses indicate that a lower ω-6/ω-3 ratio may be associated with a reduced risk of lung adenocarcinoma, suggesting a potential protective role of increased ω-3 intake in modulating inflammatory and proliferative pathways involved in lung carcinogenesis [41].

Clinical studies have demonstrated beneficial metabolic and inflammatory effects of ω-3 supplementation in patients with lung cancer. In a randomized double-blind trial including 60 patients with lung cancer, supplementation with EPA (1.6 g/day) and DHA (0.8 g/day) for 12 weeks resulted in improved nutritional parameters compared with placebo. Patients receiving ω-3 fatty acids demonstrated significantly higher body weight and serum albumin levels, accompanied by reductions in inflammatory markers such as C-reactive protein (CRP) and tumor necrosis factor-α (TNF-α). Similar results have been reported in randomized studies involving patients with stage II–III non-small cell lung cancer receiving adjuvant therapy, where ω-3 supplementation was associated with improvements in hemoglobin and albumin concentrations and significant reductions in inflammatory markers, including CRP, interleukin-6 (IL-6), and TNF-α [42].

An important aspect of ω-3 fatty acids in oncology is their potential to preserve skeletal muscle mass and prevent cancer-related cachexia. Loss of muscle mass is common among patients with lung cancer and represents an independent prognostic factor associated with worse clinical outcomes and increased risk of postoperative complications. Mechanistically, muscle catabolism in cancer is largely driven by chronic inflammation and activation of proteolytic pathways, particularly the ubiquitin–proteasome system. EPA has been shown to inhibit activation of this pathway by suppressing pro-inflammatory cytokine production and modulating NF-κB signaling. In addition, ω-3 fatty acids may stimulate muscle protein synthesis through modulation of the mTOR signaling pathway [43].

In clinical practice, ω-3 fatty acids are commonly administered as part of specialized immunonutrition formulas used in perioperative care. In many clinical trials involving cancer patients, supplementation typically includes approximately 1–2 g/day of EPA and 0.5–1 g/day of DHA, often initiated 5–10 days before surgery and in some protocols continued during the postoperative period [44].

Available evidence indicates that ω-3 fatty acids are generally safe and well-tolerated when used in recommended doses. According to the European Food Safety Authority, combined intake of EPA and DHA up to 5 g/day is considered safe for adults. However, higher doses may increase bleeding tendency, and caution is advised in patients receiving anticoagulant or antiplatelet therapy or in those with bleeding disorders [24]. The use of recommended doses in surgical patients should not significantly increase the risk of bleeding; however, studies in this area remain limited.

Concerns have occasionally been raised regarding the potential influence of ω-3 fatty acids on tumor biology because of their ability to modulate cell membrane composition, inflammatory signaling, and immune responses [21]. However, current experimental and clinical evidence does not support a cancer-promoting effect of perioperative ω-3 fatty acid supplementation. On the contrary, ω-3 fatty acids have been shown to attenuate chronic inflammation, modulate NF-κB and STAT3 signaling, and may contribute to the preservation of skeletal muscle mass and nutritional status in patients with cancer. Nevertheless, long-term oncological outcomes have not been systematically evaluated in the context of perioperative immunonutrition, and further studies are warranted [22].

### 3.3. Glutamine

Glutamine is the most abundant amino acid in human plasma and skeletal muscle and plays a crucial role in nitrogen transport, immune regulation, and maintenance of intestinal barrier integrity. Under normal physiological conditions, glutamine is synthesized endogenously in sufficient amounts; however, during periods of metabolic stress such as surgery, trauma, or severe illness, glutamine becomes a conditionally essential amino acid because endogenous production may not meet increased metabolic demands [26].

Surgical stress is associated with a rapid decline in plasma and muscle glutamine concentrations. Reduced glutamine availability may impair immune cell function, increase susceptibility to infections, and delay postoperative recovery. Glutamine serves as an essential metabolic substrate for rapidly proliferating cells, including lymphocytes, macrophages, and enterocytes. It supports lymphocyte proliferation, cytokine production, and macrophage phagocytic activity. In addition, glutamine contributes to the synthesis of glutathione, a key intracellular antioxidant that protects cells from oxidative stress during the inflammatory response to surgery [25,26,28,38].

In perioperative nutrition protocols, glutamine is most commonly administered at doses of approximately 0.3–0.5 g/kg/day, usually in the form of intravenous glutamine dipeptide or as part of enteral nutrition formulas. Supplementation is typically initiated in the preoperative period and may be continued during the early postoperative phase, particularly in patients at risk of malnutrition [12,28].

Short-term perioperative glutamine supplementation is generally considered safe, although caution is recommended in patients with severe renal or hepatic dysfunction, where amino acid metabolism may be impaired. Additionally, current clinical guidelines suggest that glutamine supplementation should be individualized and integrated into comprehensive nutritional management rather than used routinely in all surgical patients [12,25].

The use of glutamine supplementation in oncology remains controversial because many tumor cells exhibit increased glutamine metabolism to support proliferation, nucleotide synthesis, redox homeostasis, and energy production. This phenomenon, often referred to as “glutamine addiction”, has raised concerns that exogenous glutamine supplementation could theoretically promote tumor growth [45]. However, current clinical evidence does not support an increased risk of cancer progression or recurrence associated with short-term perioperative glutamine supplementation. Instead, available studies suggest that glutamine primarily supports rapidly proliferating normal tissues, including enterocytes and immune cells, thereby preserving intestinal barrier integrity, enhancing immune function, and reducing postoperative complications. Nevertheless, data regarding long-term oncological outcomes remain limited, and further studies are required to establish the long-term safety of glutamine supplementation in patients undergoing cancer surgery [46].

In the context of lung cancer surgery, glutamine supplementation may support perioperative recovery by improving nitrogen balance, supporting immune function, and enhancing antioxidant defense mechanisms. These effects may contribute to improved wound healing and reduced risk of postoperative infectious complications [25,28].

### 3.4. Nucleotides

Nucleotides are low-molecular-weight molecules that serve as the structural components of DNA and RNA and play essential roles in cellular metabolism, energy transfer, and intracellular signaling. Under physiological conditions, nucleotides can be synthesized endogenously through de novo pathways and salvage mechanisms. However, during periods of increased metabolic stress, such as major surgery, trauma, infection, or cancer, the demand for nucleotides may exceed the body’s capacity for endogenous synthesis [30].

Dietary nucleotides may therefore become important for supporting tissues with high cellular turnover, particularly immune cells and intestinal epithelial cells. The immunomodulatory effects of nucleotide supplementation are primarily related to their role in supporting lymphocyte proliferation, enhancing macrophage and neutrophil function, and regulating cytokine production. Rapidly proliferating immune cells require sufficient nucleotide availability for nucleic acid synthesis during clonal expansion and immune activation [30,47].

Experimental studies have demonstrated that nucleotide supplementation may enhance T-lymphocyte proliferation, increase natural killer (NK) cell activity, and improve the balance between CD4^+^ and CD8^+^ lymphocytes. In addition, nucleotides may contribute to maintaining intestinal mucosal integrity, which is particularly important in preventing bacterial translocation and reducing postoperative infectious complications [29].

In clinical practice, nucleotides are typically provided as part of combined immunonutrition formulas, together with arginine and ω-3 fatty acids. These nutrients are thought to act synergistically to support immune function and modulate inflammatory responses during the perioperative period. Clinical studies evaluating immunonutrition formulas containing nucleotides have demonstrated reductions in postoperative infectious complications and improved recovery in surgical populations, although the specific contribution of nucleotides alone remains difficult to isolate [48].

The amount of nucleotides included in immunonutrition formulas is usually approximately 0.3–0.5 g per serving, administered two to three times daily during the 5–10 days preceding surgery as part of oral nutritional supplementation [12].

Nucleotides administered within standard immunonutrition formulations are generally considered safe, and no specific adverse effects have been consistently reported in perioperative studies. Nevertheless, their use should be integrated into structured nutritional support protocols and tailored to the patient’s overall clinical condition [12].

### 3.5. Gut Microbiota and Other Immunomodulatory Nutrients

Emerging evidence suggests that the gut microbiota plays an important role in regulating systemic immunity, inflammation, and antitumor immune responses. Alterations in gut microbial composition (dysbiosis) have been associated with lung cancer development, impaired immune homeostasis, and reduced response to anticancer therapies through the gut–lung axis. Although clinical evidence in patients undergoing lung cancer surgery remains limited, nutritional interventions capable of modulating the gut microbiota may represent a promising adjunct to perioperative care [49].

In addition to the classical immunonutrients (arginine, glutamine, ω-3 fatty acids, and nucleotides), prebiotic compounds such as fructooligosaccharides (FOS) and dietary fiber have been investigated for their immunomodulatory properties. By promoting the growth of beneficial gut bacteria and increasing the production of short-chain fatty acids, particularly butyrate, these nutrients contribute to the maintenance of intestinal barrier integrity, modulation of inflammatory responses, and regulation of both innate and adaptive immunity. However, evidence supporting their routine perioperative use in thoracic oncology is currently insufficient, and further studies are warranted [27,50].

### 3.6. Clinical Evidence from Other Surgical Oncology Settings

Although the biological effects of individual immunonutrients have been extensively investigated, clinical evidence has been derived predominantly from studies evaluating perioperative immunonutrition formulas combining arginine, ω-3 fatty acids, nucleotides, and, less frequently, glutamine. Consequently, the clinical benefits observed cannot be attributed to a single nutrient but rather to the synergistic effects of multimodal immunonutrition [34].

Most available evidence originates from patients undergoing major gastrointestinal oncological surgery. Meta-analyses of randomized controlled trials in colorectal, gastric, pancreatic, and upper gastrointestinal surgery have consistently demonstrated that perioperative immunonutrition reduces postoperative infectious complications, shortens hospital stay, and accelerates recovery compared with standard nutritional support [28,51]. Several studies have reported improvements in adaptive immune function, reflected by restoration of the CD4^+^/CD8^+^ T-cell ratio, enhanced lymphocyte proliferation, and attenuation of postoperative inflammatory responses [51].

Clinical studies have likewise demonstrated metabolic benefits associated with immunonutrition. In malnourished patients undergoing abdominal surgery, perioperative glutamine supplementation improved antioxidant capacity, increased the reduced-to-oxidized glutathione (GSH/GSSG) ratio, and was associated with higher postoperative serum albumin concentrations. Meta-analyses have further suggested improvements in postoperative nitrogen balance together with reductions in infectious complications and hospital length of stay following glutamine-enriched nutritional support [25,28].

Overall, evidence from gastrointestinal surgical oncology strongly supports the role of perioperative immunonutrition in improving postoperative outcomes. Although these findings provide a strong rationale for perioperative immunonutrition in surgical oncology, evidence specific to lung cancer surgery remains relatively scarce. The currently available studies in thoracic oncology are therefore discussed separately in the following section [8,10,51].

## 4. Benefits of Immunonutrients in Lung Cancer Surgery

Several clinical studies have investigated the potential benefits of perioperative immunonutrition in patients undergoing lung cancer surgery. Although the number of studies specifically conducted in thoracic oncology remains limited, available evidence suggests that immunonutritional supplementation may improve nutritional status, modulate inflammatory responses, and reduce the incidence of postoperative complications [8,9,10,52].

In the study conducted by Nannoni et al., patients undergoing elective thoracic surgery received preoperative immunonutrition enriched with immunomodulating nutrients. No significant differences were observed between the immunonutrition and control groups regarding the duration of chest drainage (4.6 ± 2.5 vs. 5.4 ± 3.8 days; *p* = 0.211), length of hospital stay (5.97 ± 2.99 vs. 6.4 ± 4.1 days; *p* = 0.849), or incidence of prolonged air leak (23.3% vs. 27%; *p* = 0.809). The only statistically significant difference was a lower rate of intensive care unit admission in the immunonutrition group (48.3% vs. 75.7%; *p* = 0.011). However, the interpretation of these findings is limited by the relatively small sample size and different group sizes [52].

Additional evidence was provided by Kaya et al., who conducted a randomized study involving 31 patients undergoing lung cancer surgery. Participants received a preoperative immune-modulating nutritional supplement for 10 days prior to surgery. The study demonstrated a lower incidence of postoperative complications in the immunonutrition group compared with the control group (6 vs. 12 patients; *p* = 0.049). Furthermore, the mean duration of chest tube drainage was significantly shorter in the intervention group (median 4 days, range 2–15 days) compared with the control group (median 6 days, range 1–42 days; *p* = 0.019). Although serum albumin levels decreased in the early postoperative period in both groups, which is expected due to the inflammatory response to surgery, patients receiving immunonutrition demonstrated improved overall clinical recovery [10]. This shorter duration of chest tube drainage demonstrated by Kaya et al. carries profound implications for modern thoracic clinical practice. Within the framework of Enhanced Recovery After Surgery (ERAS) pathways, early chest tube removal directly facilitates immediate postoperative mobilization and minimizes chest wall pain, thereby synergizing with multimodal rehabilitation strategies to accelerate functional recovery [11].

Shoji et al. evaluated the effects of short-term preoperative immunonutritional support administered for 7–14 days before elective thoracic surgery. The authors reported significant improvements in both the Prognostic Nutritional Index (PNI) and the Geriatric Nutritional Risk Index (GNRI) in the intervention group, indicating improved preoperative nutritional status and immune competence. These indices have been shown to correlate with postoperative outcomes and long-term prognosis in patients with lung cancer. However, the intervention itself was limited to a short preoperative period, and the study did not evaluate whether these improvements translated into long-term oncological outcomes [9].

Additional observational evidence supports the role of immunonutrition in surgical oncology. In a large retrospective study including 620 patients undergoing major abdominal cancer surgery, preoperative immunonutrition administered for 5 days was associated with reduced infectious complications and decreased need for intensive care support, mechanical ventilation, and vasopressor therapy. Although these results originate from abdominal surgery, they support the concept that perioperative immunonutrition may beneficially influence surgical outcomes in oncologic populations [53].

Further evidence from studies involving patients undergoing lung cancer surgery has demonstrated improvements in nutritional and inflammatory indices following perioperative immunonutritional supplementation. In one study, patients received oral immunonutrition twice daily for 10 days before surgery and for five days postoperatively. Laboratory parameters including serum albumin, lymphocyte count, neutrophil count, and platelet count were measured, allowing calculation of the Prognostic Nutritional Index (PNI) and the Systemic Immune-Inflammation Index (SII). Although the PNI decreased and the SII increased in the immediate postoperative period, reflecting the physiological inflammatory response to surgery, patients receiving immunonutrition demonstrated better postoperative nutritional status. In the postoperative period, 85% of patients in the treatment group were well nourished, while only a minority had mild-to-moderate malnutrition. Moreover, the Patient-Generated Subjective Global Assessment (PG-SGA) score was significantly better in the intervention group compared with the control group (*p* = 0.046) [8]. These clinical findings underscore the critical importance of proper patient selection in thoracic oncology. Given the high prevalence of cancer cachexia and sarcopenia in non-small cell lung cancer, unselected immunonutrition may dilute visible clinical benefits [16,54]. The positive shifts in baseline indices such as PNI and GNRI strongly suggest that the therapeutic window for immunonutrition is widest in patients presenting with established malnutrition, elevated systemic inflammatory markers (e.g., neutrophil-to-lymphocyte ratio), or significant muscle wasting who face the highest risk of catabolism following surgical trauma [55,56].

Overall, current evidence suggests that perioperative immunonutrition may improve nutritional and immunological status in patients undergoing lung cancer surgery and may reduce postoperative complications and improve recovery [9,10,52]. However, the available studies are relatively small and heterogeneous, and further large, well-designed randomized clinical trials are required to establish the optimal composition, timing, and duration of immunonutrition in thoracic surgical oncology [57]. Despite these promising findings, the currently available evidence regarding perioperative immunonutrition in lung cancer surgery remains limited and should be interpreted with caution. Most studies included relatively small patient cohorts, which substantially limits statistical power and the generalizability of the results. In several trials, patient populations were heterogeneous with respect to tumor stage, baseline nutritional status, comorbidities, and perioperative risk profiles, potentially influencing postoperative outcomes independently of nutritional intervention. Moreover, important surgical variables were often insufficiently reported, including the extent of pulmonary resection and the surgical approach used, such as minimally invasive video-assisted thoracoscopic surgery (VATS) versus open thoracotomy. Given that minimally invasive techniques are associated with reduced surgical stress and inflammatory response, the potential benefits of immunonutrition may differ depending on the operative approach [8,9,10,52].

Another important limitation is the lack of standardization regarding the composition, timing, duration, and route of immunonutritional supplementation across studies. Different formulations enriched with arginine, omega-3 fatty acids, nucleotides, or glutamine were used, making direct comparison between studies difficult [9]. In addition, most available studies focused primarily on short-term postoperative outcomes, while data regarding long-term survival, recurrence rates, quality of life, functional recovery, and preservation of skeletal muscle mass remain scarce [58].

Furthermore, as the treatment paradigm for early and locally advanced NSCLC rapidly shifts toward neoadjuvant and adjuvant chemo-immunotherapy (e.g., immune checkpoint inhibitors targeting PD-1/PD-L1), the intersection between nutritional immunology and oncological therapies demands urgent exploration. Immunonutrients like arginine and omega-3 fatty acids are known to modify the tumor microenvironment and functional phenotypes of T lymphocytes and natural killer cells [59]. Whether perioperative metabolic modulation can synergize with or affect the efficacy of modern immunotherapy remains a crucial, unanswered question for future thoracic oncology trials.

Several clinical studies have evaluated the impact of perioperative immunonutrition on postoperative outcomes in patients undergoing lung cancer surgery and other major oncologic procedures. A summary of the most relevant studies is presented in Table 2.

## 5. Immunonutrition Protocols in the Perioperative Period: Current Guideline Recommendations

Current international guidelines emphasize the importance of appropriate perioperative nutritional management in patients undergoing major oncologic surgery, including lung cancer resection. Optimization of nutritional status before surgery is considered a key component of perioperative care, as malnutrition and cancer-related cachexia are associated with increased postoperative morbidity, prolonged hospital stays, and poorer overall outcomes [54,60,61].

According to the guidelines of the European Society for Clinical Nutrition and Metabolism (ESPEN), all surgical patients should be routinely screened for malnutrition using validated tools. Patients identified as being at nutritional risk should receive oral or enteral nutritional support prior to surgery. In patients undergoing major cancer surgery, particularly those with established malnutrition or at high metabolic risk, the use of specialized immunonutrition formulas enriched with arginine, omega-3 fatty acids, and nucleotides is recommended. Preoperative administration of immunonutrition for approximately 5–7 days is suggested to improve immune competence and potentially reduce postoperative infectious complications. In severely malnourished patients, a longer period of preoperative nutritional support may be necessary before surgical intervention is undertaken [12].

Similarly, recommendations from the American Society for Enhanced Recovery (ASER) and the Perioperative Quality Initiative (POQI) highlight the importance of perioperative nutritional optimization as part of enhanced recovery pathways. These guidelines recommend that patients at nutritional risk receive oral or enteral nutritional supplementation, including immunomodulating formulas, for 5–10 days before surgery when feasible. Nutritional interventions should be integrated into a broader multimodal perioperative strategy, including early mobilization, optimized analgesia, minimally invasive surgical techniques, and early postoperative feeding [62].

In thoracic surgery specifically, Enhanced Recovery After Surgery (ERAS) protocols for lung resection also recognize the importance of preoperative nutritional assessment and optimization. The ERAS Society guidelines for lung surgery recommend systematic screening for malnutrition in patients undergoing pulmonary resection and emphasize that patients with impaired nutritional status should receive targeted nutritional support before surgery. Although thoracic-specific guidelines do not uniformly mandate immunonutrition for all patients, they acknowledge that specialized nutritional formulas may be beneficial in individuals with malnutrition, sarcopenia, or significant metabolic stress [11].

Postoperatively, guidelines recommend reassessment of nutritional status using a combination of clinical evaluation, anthropometric measurements, and validated screening tools such as the Patient-Generated Subjective Global Assessment (PG-SGA) or the Prognostic Nutritional Index (PNI). These assessments allow clinicians to adjust nutritional interventions during the recovery period and to ensure adequate energy and protein intake during rehabilitation [11,12,62].

Beyond nutritional screening tools, recent ESPEN recommendations emphasize the assessment of body composition. Bioelectrical impedance analysis (BIA), computed tomography-derived skeletal muscle index, and muscle strength assessment may improve identification of sarcopenia and cachexia, allowing better selection of patients who are most likely to benefit from perioperative immunonutrition [12].

Overall, current clinical guidelines support the integration of perioperative nutritional strategies, including immunonutrition, into comprehensive perioperative care for patients undergoing major oncologic surgery. The primary goals of such interventions are to improve immune competence, reduce postoperative infectious complications, preserve skeletal muscle mass, and facilitate faster functional recovery following lung cancer surgery. Although current thoracic surgery guidelines strongly support perioperative nutritional assessment and intervention in patients undergoing lung cancer surgery, the clinical significance of immunonutrition remains uncertain due to the limited number and heterogeneity of thoracic-specific clinical studies [12,13].

## 6. Discussion

Perioperative management in lung cancer surgery has increasingly shifted toward multimodal ERAS-based pathways, in which nutritional optimization is considered an integral component of perioperative care [12,13,15]. Within this framework, immunonutrition has emerged as a biologically plausible intervention to attenuate surgical stress and modulate immune dysfunction. Despite a strong mechanistic rationale for substrates such as arginine, omega-3 fatty acids, glutamine, and nucleotides, translating these effects into consistent clinical benefit in thoracic oncology remains challenging. The current body of thoracic-specific evidence suggests a disconnect between improvements in nutritional or inflammatory surrogate markers and hard clinical outcomes [16]. Studies by Shoji et al. and Gunsel et al. demonstrate consistent improvements in indices such as PNI, GNRI, and PG-SGA following preoperative immunonutrition. While these markers are clinically relevant indicators of nutritional status, their ability to predict meaningful postoperative outcomes remains imperfect. Consequently, improvements in surrogate nutritional endpoints should not be automatically interpreted as evidence of improved surgical outcomes. These findings indicate that short-term nutritional intervention can meaningfully modify baseline metabolic status prior to surgery. However, whether these improvements translate into reduced postoperative morbidity remains unclear and appears to depend heavily on study design, patient selection, and surgical context. This discrepancy becomes particularly evident when comparing clinical outcomes across studies [8,9]. Kaya et al. reported a reduction in overall postoperative complications and shorter chest tube duration following a 10-day preoperative immunonutrition regimen, suggesting a clinically relevant effect on recovery [10]. In contrast, Nannoni et al. observed no significant differences in most perioperative endpoints, apart from ICU admission rates. These divergent findings likely reflect differences in sample size, patient population heterogeneity, and variability in surgical technique [52]. An important limitation of the current evidence is the relatively short duration of immunonutrition interventions and follow-up. In most randomized clinical trials, immunonutrition is administered for only 5–14 days during the perioperative period, and the primary outcomes are limited to postoperative complications, inflammatory markers, and short-term recovery. Consequently, the long-term effects of perioperative immunonutrition on oncological outcomes, including tumor recurrence, disease-free survival, and overall survival, remain largely unknown. Although available clinical studies have not demonstrated evidence of increased tumor progression associated with perioperative immunonutrition, they were not designed or powered to evaluate long-term oncological safety. Future prospective studies with extended follow-up are therefore required to determine whether perioperative immunonutrition influences long-term cancer outcomes and to further establish its safety profile in patients undergoing lung cancer surgery [57]. In addition, heterogeneous inclusion of patients undergoing both video-assisted thoracoscopic surgery (VATS) and open thoracotomy complicates interpretation [63]. Since minimally invasive approaches are associated with a substantially reduced inflammatory and stress response, the marginal benefit of immunonutrition may be less detectable in such cohorts. Another important consideration is patient selection. The available evidence suggests that the clinical impact of immunonutrition is likely not uniform across all surgical candidates. Patients with pre-existing sarcopenia, cancer-related cachexia, or elevated systemic inflammatory burden appear more likely to benefit than well-nourished individuals [55,60]. In this context, the biological effects of substrates such as glutamine may be particularly relevant during one-lung ventilation, where ischemia–reperfusion injury and oxidative stress contribute to postoperative pulmonary complications [25,26]. Future studies should move beyond treating surgical patients as a homogeneous population and instead focus on biologically and nutritionally vulnerable subgroups. Patients with sarcopenia, cachexia, frailty, or elevated systemic inflammation may derive greater benefit from perioperative immunonutrition than metabolically preserved individuals [56]. However, this remains largely speculative in the absence of robust thoracic-specific randomized data. Importantly, currently available evidence suggests that immunonutrition exerts predominantly qualitative rather than quantitative effects on innate immunity, improving immune cell function without consistently altering circulating leukocyte subsets. This observation is consistent with clinical studies demonstrating enhanced phagocytic activity, oxidative burst, and modulation of inflammatory cytokine production despite the absence of substantial changes in peripheral leukocyte counts. An additional and still insufficiently explored dimension is the interaction between immunonutrition and modern systemic oncologic therapies. The treatment landscape for resectable non-small cell lung cancer is rapidly evolving toward neoadjuvant chemo-immunotherapy regimens incorporating immune checkpoint inhibitors [59]. Given that immunonutrients can influence T-cell activity, macrophage polarization, and NK cell function, they may interact with immunotherapy-induced immune modulation. Whether such effects are synergistic, neutral, or potentially counterproductive remains unknown and represents an important area for future investigation [48]. Overall, while current guidelines support perioperative nutritional optimization in patients undergoing lung cancer surgery, the specific role of immunonutrition remains incompletely defined. Small sample sizes, heterogeneity in nutritional formulations and timing, and a lack of standardized clinical endpoints limit the existing evidence base. Moreover, long-term outcomes such as survival, recurrence, functional recovery, and quality of life have been insufficiently studied. Future research should therefore focus on large, multicenter randomized controlled trials with standardized immunonutrition protocols and clearly defined patient stratification criteria. Particular attention should be given to high-risk subgroups, including patients with sarcopenia, malnutrition, or a high inflammatory burden [8,9,10,52]. Additionally, the integration of immunonutrition into contemporary ERAS pathways, in combination with modern immunotherapies, should be prospectively evaluated to clarify its clinical value in thoracic oncology [13,63]. Perioperative immunonutrition represents a promising and evolving strategy in the comprehensive care of patients undergoing lung cancer surgery. Taken together, the available evidence suggests that perioperative immunonutrition may improve nutritional and inflammatory parameters in selected patients undergoing lung cancer surgery. However, the impact of these changes on clinically meaningful outcomes remains uncertain because of the limited number of thoracic-specific studies and substantial heterogeneity across existing trials [60].

## 7. Conclusions

Despite these promising results, small sample sizes and heterogeneity in clinical protocols limit many existing studies. Therefore, while current guidelines support the use of immunonutrition in patients at nutritional risk, there is a clear need for large-scale, multicenter randomized controlled trials specifically focused on thoracic oncology. Future research must aim to define the optimal composition, precise timing, and duration of nutritional support, as well as identify specific patient subgroups that would derive the greatest benefit from personalized immunonutritional interventions.

## Figures and Tables

**Table 1:** Summary of perioperative immunonutrients used in clinical practice.

Nutrient	Typical Dose in Clinical Studies	Timing of Administration	Main Clinical Effects	Safety Considerations
Arginine	~0.5–1.5 g/kg/day (within immunonutrition formulas) [17]	5–10 days preoperatively, sometimes continued postoperatively [18]	Enhanced NK and T-cell activity, improved immune recovery, reduced postoperative complications [19]	Generally safe for short-term perioperative use; caution in severe renal or hepatic insufficiency [20]
Omega-3 fatty acids (EPA/DHA)	~1–2 g EPA + 0.5–1 g DHA/day [21]	5–10 days preoperatively; sometimes continued postoperatively [21]	Reduced inflammatory markers, improved nutritional status, preservation of skeletal muscle mass [22,23]	Safe up to ~5 g/day EPA + DHA; caution with anticoagulant therapy [24]
Glutamine	~0.3–0.5 g/kg/day [25]	Preoperative and/or early postoperative period [26]	Improved nitrogen balance, antioxidant capacity, and immune cell function [27]	Use cautiously in severe renal or hepatic failure [28]
Nucleotides	~0.3–0.5 g per serving (within formulas) [29]	Typically 5–10 days before surgery [30]	Support lymphocyte proliferation and intestinal barrier function [29]	Generally safe as part of immunonutrition formulas [30]

Abbreviations: EPA, eicosapentaenoic acid; DHA, docosahexaenoic acid; NK, natural killer.

**Table 2:** Overview of clinical evidence and perioperative outcomes associated with immunonutrition.

Study	Design	*N*	Population	Intervention	Main Outcomes	Limitations	Duration
Nannoni et al. [52]	Prospective comparative study	59	Elective thoracic surgery	Preoperative immunonutrition formula containing arginine, ω-3 fatty acids, and nucleotides	↓ ICU admission	small sample size, non-randomized groups, different group sizes	Not reported
Kaya et al. [10]	Randomized controlled study	31	patients undergoing lung cancer surgery	Preoperative immunomodulating nutritional supplementation for 10 days before surgery	↓ complications, ↓ drainage time	small sample size, single center	10 days
Shoji et al. [9]	Prospective cohort study	39	Elective thoracic surgery	Short-term preoperative immunonutrition (7–14 days)	↑ PNI, GNRI	small sample size, heterogeneous supplement types, no blinding	7 to 14 days
Gunsel et al. [8]	Prospective randomized controlled trial	70	Lung cancer resection	Oral immunonutrition twice daily for 10 days before surgery and 5 days after surgery	improved PG-SGA	small sample size, heterogeneity in patient baseline nutritional status	10 days before the surgery and 5 days after the surgery

Abbreviations: GNRI, Geriatric Nutritional Risk Index; ICU, intensive care unit; PG-SGA, Patient-Generated Subjective Global Assessment; PNI, Prognostic Nutritional Index; ↑ Increase; ↓ decrease.

## Data Availability

No new data were created or analyzed in this study. Data sharing is not applicable to this article.

## Abbreviations

The following abbreviations are used in this manuscript:

BIA	Bioelectrical Impedance Analysis
BMI	Body Mass Index
CRP	C-reactive Protein
CT	Computed Tomography
DHA	Docosahexaenoic Acid
EPA	Eicosapentaenoic Acid
ERAS	Enhanced Recovery After Surgery
ESPEN	European Society for Clinical Nutrition and Metabolism
FOS	Fructooligosaccharides
GLIM	Global Leadership Initiative on Malnutrition
iNOS	Inducible Nitric Oxide Synthase
IL	Interleukin
MUST	Malnutrition Universal Screening Tool
NF-κB	Nuclear Factor Kappa B
NK	Natural Killer (cell)
NLR	Neutrophil-to-Lymphocyte Ratio
NSCLC	Non-Small Cell Lung Cancer
PG-SGA	Patient-Generated Subjective Global Assessment
RCT	Randomized Controlled Trial
ROS	Reactive Oxygen Species
SCFA	Short-Chain Fatty Acids
SMI	Skeletal Muscle Index
TGF-β	Transforming Growth Factor Beta
Th	T Helper (cell)
TLR	Toll-Like Receptor
TNF-α	Tumor Necrosis Factor Alpha
Treg	Regulatory T Cell
